# Open globe injury and intraocular foreign body following crossbow-related penetrating ocular trauma

**DOI:** 10.1016/j.ajoc.2022.101441

**Published:** 2022-02-23

**Authors:** Shawn Gulati, Kurt A. Hanebrink, Michael Henry, Monique Munro, R.V. Paul Chan, Deepak P. Edward

**Affiliations:** Illinois Eye and Ear Infirmary, Department of Ophthalmology and Visual Sciences, University of Illinois at Chicago, USA

**Keywords:** Ocular trauma, Crossbow, Bow, Arrow, Intraocular foreign body

## Abstract

**Purpose:**

To describe a case of a penetrating ocular trauma and plastic intraocular foreign body (IOFB), undetected on preoperative imaging.

**Observations:**

We present the findings of a 40-year-old male who sustained an open globe injury and IOFB composed of plastic following crossbow-related trauma. Preoperative detection of the IOFB was unsuccessful on clinical exam, computed tomography (CT) and ultrasonography. During extraction of the traumatic cataract, an intralenticular IOFB was discovered and removed through an enlarged limbal incision. Postoperative review revealed that a fragmented plastic “nock”, from the crossbow arrow bolt, was the likely IOFB source. The bolt was produced by injection molding which may lead to trapped gas within the plastic, causing radiolucency on CT.

**Conclusions and importance:**

Radiolucent plastic warrants consideration on the differential diagnosis when intraocular gas is noted on computed tomography following penetrating ocular trauma. Multimodal imaging should be considered if IOFB is suspected and not detected by CT.

## 1 Introduction

The management of open globe injuries with intraocular foreign bodies (IOFB) is often challenging. Many cases have complicated clinical courses and necessitate multiple patient visits and surgeries with coordinated care among several ophthalmologic services.^1^ In the setting of an open globe injury and ocular trauma, accurate detection of IOFB is critical for appropriate surgical planning. Herein, we describe a case in which IOFB detection was difficult due to its composition and location. We also present management strategies employed in the care of a penetrating ocular trauma patient.

### 1.1 Case report

A 40-year-old male in good health presented to an outside hospital after sustaining trauma to the left eye. He was releasing the tension of a crossbow with a decocking bolt, when a projectile object flew upward hitting him in the left eye. He then experienced a sudden decrease in vision. At that point, the nature of the object that hit the eye was uncertain.

**Fig. 1:**
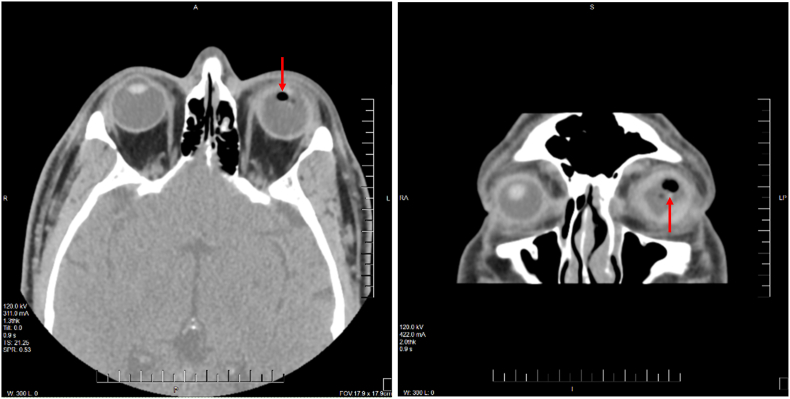
Computed tomography orbits without intravenous contrast. A. Axial cut soft tissue window demonstrating intralenticular “air” (arrow) in the left eye. B. Coronal cut soft tissue window demonstrating intralenticular “air” (arrow) in the left eye. The term “air” is noted in quotations because the trapped gas within the IOFB limits the plastic component from being seen on CT.

On examination at the outside hospital, left eye visual acuity (VA) was hand motion and intraocular pressure (IOP) was not obtained due to open globe injury. On slit lamp exam, there was a full thickness linear corneal laceration extending from the inferior cornea toward the temporal cornea with iris tissue incarcerated in the wound. A traumatic cataract with anterior capsule violation was also noted. Computed tomography (CT) scan of the orbits without intravenous contrast demonstrated “scattered air” at the level of the lens as well as suspected suprachoroidal or vitreous hemorrhage (Fig. 1). There were no radiopaque foreign bodies noted. The corneal laceration was repaired with several interrupted 10-0 nylon sutures with repositioning of the iris at the referring facility.

At postoperative day 1 visit at the outside institution, the nasal edge of the corneal laceration was Seidel positive with a shallow anterior chamber (AC). IOP was 14 mm Hg and a bandage contact lens was placed. Immediately following this clinic visit, the patient returned home and presented to our institution the same day for continued management. On exam, the AC was flat and there was a brisk wound leak noted from the nasal edge of the corneal laceration, which was repaired.

**Fig. 2:**
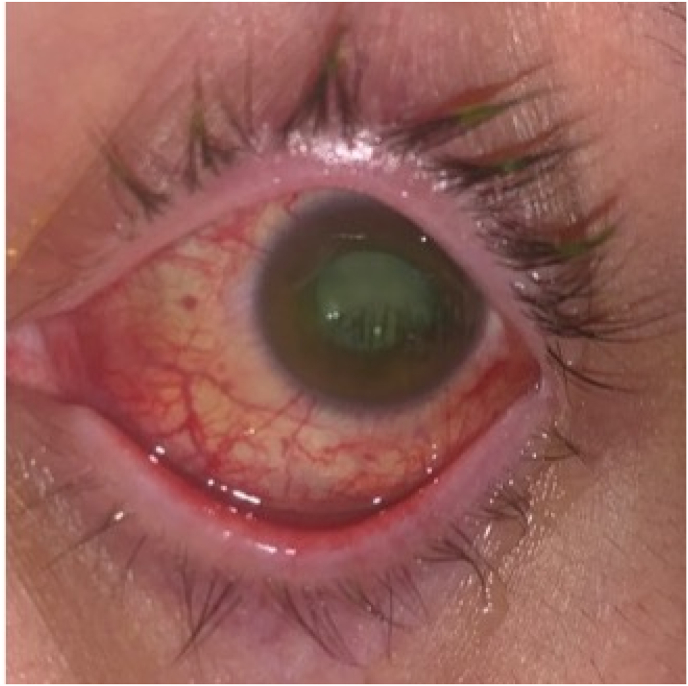
Left eye external photograph taken prior to cataract extraction demonstrating corneal laceration repaired with several interrupted 10-0 nylon sutures, shallow anterior chamber, and fluffy white lenticular material protruding through violated anterior capsule. No intraocular foreign body is appreciated.

On the following day, the IOP was elevated to 55 mmHg, which was not responsive to topical glaucoma medications and oral acetazolamide. The AC was shallow with iris anteriorly displaced from the traumatic cataract with white lenticular material protruding from the lens through the violated anterior capsule (Fig. 2). No phacodonesis or zonular dialysis was noted. The mechanism of glaucoma was felt to be mixed phacomorphic and lens particle mechanisms. B-scan ultrasonography was performed which showed the crystalline lens in normal position without abnormal echoic activity with no intralenticular or posterior segment foreign body and or additional retinal or choroidal pathology.

Following perioperative intravenous mannitol, extracapsular cataract surgery was planned with possible IOL placement with retina service on standby should posterior capsule violation be encountered.

**Fig. 3:**
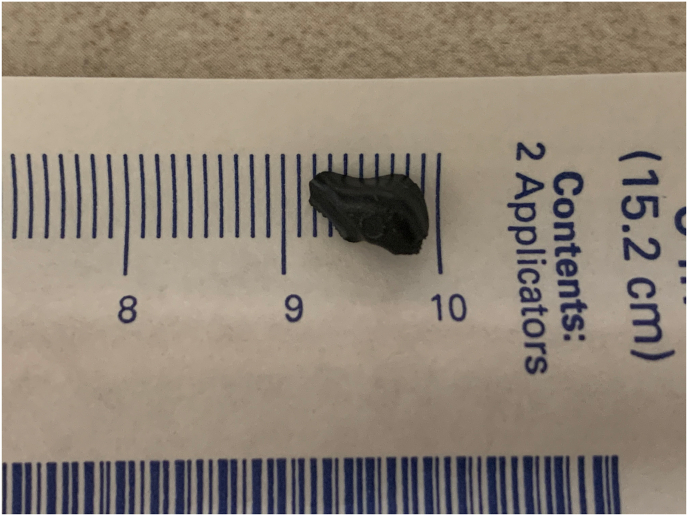
Left eye non-metallic intraocular foreign body measuring approximately 7 mm.

After initial standard steps of cataract surgery were performed, a Simcoe canula was used to gently aspirate the majority of the cortex, leaving one large piece which was initially believed to be nuclear material because of the dark color and lamination, and the lack of foreign body noted on both CT and B-scan (UBM was not performed as IOFB was not detected with other methods). During attempted phacoemulsification, the fragment descended into the anterior vitreous through a posterior capsular defect. A 25G pars plana vitrectomy (PPV) was initiated and upon debridement of anterior vitreous, it was apparent that this fragment was a foreign body with better visualization of its dark color and composition. Due to the size of the fragment, removal was pursued through an enlarged limbal corneal incision. The limbal corneal incision was enlarged to 8 mm, and a lens loop and foreign body forceps were used to remove the foreign body with concurrent pressure placed 180° away from the corneal incision. The foreign body was black and non-metallic (Fig. 3). All wounds were closed and the patient was left aphakic.

Briefly, the postoperative course included choroidal detachment which progressively resolved over 6 weeks. The patient developed a macula-on retinal detachment 2 months following the initial surgery, which was repaired with PPV, endolaser and left under silicone oil. The case was combined with penetrating keratoplasty at that time as well. After postoperative IOP spikes refractory to medical management, limited diode cyclophotocoagulation was performed which has successfully controlled IOP over the subsequent 2 months At most recent follow up 2 months post-PPV for RD repair, VA measured 20/300 with pinhole improvement to 20/50 without correction, IOP was 19 mm Hg and the penetrating keratoplasty was healing well.

## 2 Discussion

**Fig. 4:**
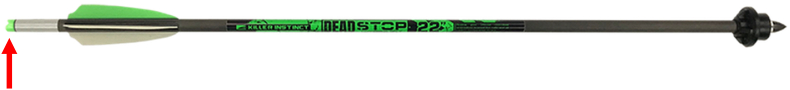
**Killer Instinct DeadStop**^**TM**^**Decocking Bolt.**^2^**The red arrow points to the “nock”, which is the suspected intraocular foreign body source in our case.** (For interpretation of the references to color in this figure legend, the reader is referred to the Web version of this article.)

**Fig. 5:**
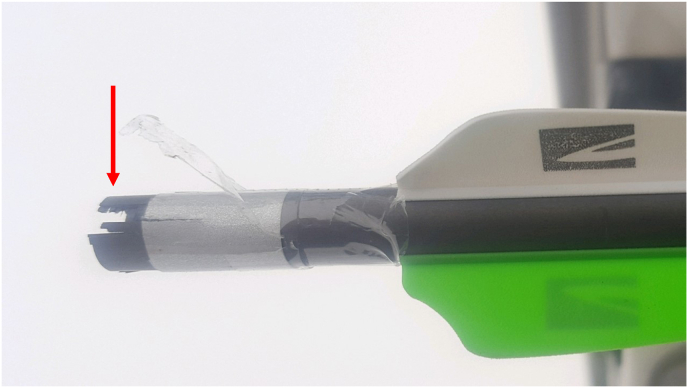
Photograph of the fragmented “nock” (arrow) from the Killer Instinct DeadStop™ Decocking Bolt used at the time of trauma.

This case highlights an unusual presentation of an intralenticular FB that was not initially identified on clinical exam and with two preoperative imaging methods, raising the question of its source and composition. Upon further questioning, the patient believed the IOFB to be from a crossbow arrow termed DeadStop™ Decocking Bolt (Killer Instinct, Windom, MN, USA) (Fig. 4). The patient has since noted that the end of the bolt, termed the “nock”, was fragmented following the incident (Fig. 5). Discussion with the manufacturing company informed us that this piece was made of polycarbonate, which is produced by injection molding. This process can lead to trapped gas (i.e. air) within the plastic, which would appear radiolucent on CT as was noted in our patient. Conversely, the other parts of the bolt, including tip, shaft and insert, are made with steel, brass, or aluminum, all of which, being metals, would appear radiopaque on CT.

Plastics such as polycarbonate have low-intensity radiopacity, but may be undetectable while surrounded by bone, muscle or air.^3^ Lakits et al. reported a case of successful detection of a plastic IOFB by CT (radiopaque within the vitreous cavity), though the exact composition and manufacturing process of the plastic was not noted.^4^ No other case reports of an intralenticular plastic IOFB were found in the literature, though Joos et al. reported a case of a plastic foam orbital foreign body masquerading as air on CT.^5^ Given the overt radiolucency of the IOFB in our case, we suspect that trapped gas within the plastic from the manufacturing process may have contributed to the CT findings.

Accurate detection of IOFBs following ocular trauma is critical for proper surgical planning. They are typically removed at the time of initial globe repair or during subsequent surgery depending on the specifics of the case. Prompt identification of an intraocular foreign body would allow for appropriate consultation of a vitreoretinal service. Clinical examination, B-scan ultrasonography and CT imaging are all helpful in detecting the presence of an IOFB. This case posed an unusual challenge in that none of these modalities picked up on the presence of a large intralenticular foreign body until it was directly visualized at the time of cataract surgery, including B scan likely due to its anterior location. Given the radiolucency seen on CT was unexpected and located anteriorly within the lens, ultrasound biomicroscopy might have been helpful in identifying the intralenticular FB preoperatively. Sensitivity and specificity of ultrasound to detect soft tissue foreign bodies have been reported as 43%–100% and 70%–100%, respectively.^6^^,^^7^ Operator experience, size/depth of foreign body, and neighboring structure echogenicity may impact the likelihood of detection.

Reports on bow and arrow injuries with retained foreign bodies are rare in the ocular region, but as illustrated by our case and others in the literature, IOFB detection can be challenging because of variable composition of the parts. O'Neill et al. reported a case of transorbital penetrating head injury with a hunting arrow, but the aluminum shaft arrow is apparent and appears radiopaque on CT.^8^ Other studies have also reported successful detection of radiopaque foreign bodies from arrow injuries with x-ray and CT.^9^^,^^10^ However, when the foreign body is radiolucent, radiographic detection falls considerably, with only 15% of wooden foreign bodies detected.^11^ Magnetic resonance imaging may be used when foreign body is suspected but involves increased time and cost burdens. Alternatively, ultrasound has advantages of being inexpensive and readily available. Robertson et al. described a case in which ultrasound was used to successfully identify a carbon-fiber foreign body from an arrow in a forearm.^12^

Overall, Rong et al. believe that ophthalmic providers must understand the limits of each imaging modality in detecting various materials, and multimodal imaging is recommended if there is suspicion of an IOFB.^13^ Thus, in patients with penetrating intraocular injury, for whom radiographic imaging is negative for foreign body yet radiolucent foreign body is suspected, multimodal imaging should be considered.

## 3 Conclusion

Accurate IOFB detection following open globe injury from penetrating trauma is crucial for proper management and surgical planning. However, the growing use of various plastics in projectile objects may pose an increasing clinical challenge as its detection is sometimes difficult. Detailed history taking, clinical examination and provider awareness of possible imaging limitations of projectile materials are critical to successfully manage these cases of complex ocular trauma. When caring for patients with prior ocular trauma where there is a high index of suspicion for an IOFB, multimodal imaging should be considered if the IOFB is not initially detected by CT.

## Patient consent

Consent to publish this case report has been obtained from the patient in writing. This report does not contain any personal identifying information.

## Patient consent

Consent to publish this case report has been obtained from the patient in writing. This report does not contain any personal identifying information.

## Declaration of competing interest

The authors declare the following financial interests/personal relationships which may be considered as potential competing interests: This report received financial support from an Unrestricted Departmental Grant from Research to Prevent Blindness (New York, NY). The authors have no conflicts of interest to declare.
